# A Comparison of Clinical Characteristics, Outcomes, and Predictors of Adverse Outcomes in Patients With Heart Failure With Preserved Versus Reduced Ejection Fraction: A Retrospective Study

**DOI:** 10.7759/cureus.111870

**Published:** 2026-07-01

**Authors:** Ali Husnain, Aliqa Fatima, Sana Saleem, Mohammad Yahya, Syeda Hadiqa Hassan, Muhammad Ammar Arif, Ali Raza

**Affiliations:** 1 Cardiology, Shalamar Hospital, Lahore, PAK; 2 Internal Medicine, Jinnah Hospital, Lahore, PAK; 3 Medical Education, Abu Umara Medical and Dental College/Green International University, Lahore, PAK; 4 Biochemistry, Queen's Medical College, Kasur, PAK; 5 Cardiology, Fatima Memorial Hospital, Lahore, PAK; 6 Emergency Medicine, Shalamar Hospital, Lahore, PAK

**Keywords:** clinical outcomes, ejection fraction, heart failure, mortality, predictors

## Abstract

Background and objective

Heart failure (HF) is a leading cause of morbidity and mortality worldwide and is broadly classified into heart failure with reduced ejection fraction (HFrEF) and heart failure with preserved ejection fraction (HFpEF), which differ in pathophysiology, clinical profile, and outcomes. This study aimed to compare the demographic characteristics, clinical profile, comorbidity burden, and short-term clinical outcomes, including in-hospital mortality, length of hospital stay, and 30-day readmission, between patients with HFpEF and HFrEF, and to identify independent predictors of adverse clinical outcomes.

Methodology

This retrospective comparative observational study was conducted at Shalamar Hospital, Lahore, Pakistan, from March 2023 to March 2026. A total of 305 patients with confirmed heart failure were included using non-probability consecutive sampling. Patients were categorized into HFrEF (left ventricular ejection fraction (LVEF) < 40%) and HFpEF (LVEF ≥ 50%) groups. Data were extracted from medical records using a structured proforma.

Results

Of 305 patients, 182 (59.7%) had HFrEF and 123 (40.3%) had HFpEF. Patients with HFpEF were significantly older and had a higher prevalence of hypertension, diabetes mellitus, and obesity (p < 0.05). HFrEF patients more commonly had ischemic heart disease and presented with more advanced symptoms (p < 0.05). In-hospital mortality was higher in HFrEF compared to HFpEF (26, 14.3% vs. 10, 8.1%), although the difference was not statistically significant (p = 0.089). HFrEF was associated with longer hospital stay (7.6 ± 3.2 vs. 5.9 ± 2.7 days, p = 0.001) and higher 30-day readmission rates (50, 27.5% vs. 23, 18.7%, p = 0.045).

Conclusions

HFrEF is associated with worse short-term clinical outcomes, including prolonged hospitalization and higher readmission rates, whereas HFpEF is associated with a greater burden of comorbid conditions.

## Introduction

Heart failure (HF) is a major global public health problem and a leading cause of morbidity, mortality, and healthcare expenditure worldwide [1]. It affects millions of individuals and is associated with frequent hospitalizations, impaired quality of life, and substantial economic burden. Despite significant advances in diagnostic and therapeutic strategies, the prognosis of HF remains poor, particularly in low- and middle-income countries where delayed diagnosis, limited access to specialized care, and inadequate healthcare resources contribute to adverse outcomes [2].

Based on left ventricular ejection fraction (LVEF), HF is broadly classified into HF with reduced ejection fraction (HFrEF), defined as LVEF < 40%, and HF with preserved ejection fraction (HFpEF), defined as LVEF ≥ 50% [3]. These phenotypes differ considerably in terms of pathophysiology, clinical characteristics, treatment response, and prognosis. HFrEF is primarily characterized by impaired systolic function resulting from myocardial injury, commonly secondary to ischemic heart disease, cardiomyopathies, or prior myocardial infarction. In contrast, HFpEF is predominantly associated with diastolic dysfunction, impaired ventricular relaxation, and increased myocardial stiffness, and is frequently observed in older adults and patients with multiple comorbidities such as hypertension, diabetes mellitus, obesity, and atrial fibrillation [4].

The management of HFrEF has evolved substantially over recent decades, with several evidence-based pharmacological therapies demonstrating significant reductions in mortality and hospitalization rates. Conversely, therapeutic options for HFpEF remain comparatively limited, and management is largely focused on symptom control and optimization of comorbid conditions [5]. Furthermore, the heterogeneous clinical presentation and complex pathophysiology of HFpEF continue to pose diagnostic and therapeutic challenges.

The underlying mechanisms of these two HF phenotypes also differ significantly. HFrEF is commonly associated with ventricular dilatation, reduced myocardial contractility, and activation of neurohormonal pathways, including the renin-angiotensin-aldosterone system and sympathetic nervous system [6,7]. In contrast, HFpEF is characterized by concentric ventricular remodeling, endothelial dysfunction, systemic inflammation, and increased myocardial stiffness. The coexistence of multiple comorbidities contributes to a complex pathophysiological environment in HFpEF and may partly explain the limited effectiveness of targeted therapies in this population [8].

Clinical outcomes in HF vary by ejection-fraction phenotype. Patients with HFrEF generally experience higher rates of cardiovascular mortality and disease progression. In contrast, patients with HFpEF often have a greater burden of non-cardiovascular comorbidities that contribute to overall morbidity and mortality [9]. Despite differences in pathophysiology, both groups experience frequent hospital readmissions, reduced exercise tolerance, and impaired quality of life, posing significant challenges to long-term disease management.

Identification of predictors of adverse outcomes is essential for risk stratification, individualized treatment planning, and optimization of healthcare resources [10]. Various clinical, biochemical, and echocardiographic parameters have been investigated as potential prognostic factors, including age, sex, BMI, renal function, natriuretic peptide levels, left atrial size, and indices of diastolic dysfunction [11]. Additionally, comorbid conditions such as hypertension, diabetes mellitus, chronic kidney disease, and atrial fibrillation have been consistently associated with poorer outcomes in patients with HF. However, the relative contribution of these predictors may differ between HFpEF and HFrEF, highlighting the need for comparative evaluation of these distinct phenotypes [12].

Given the increasing prevalence of HF and the evolving understanding of its phenotypic subtypes, a comparative assessment of clinical characteristics, outcomes, and prognostic factors in patients with HFpEF and HFrEF may provide valuable insights for improving patient management and risk stratification. This study aimed to compare the demographic characteristics, clinical profile, comorbidity burden, and short-term clinical outcomes, including in-hospital mortality, length of hospital stay, and 30-day readmission, between patients with HFpEF and HFrEF, and to identify independent predictors of adverse clinical outcomes.

## Materials and methods

Study design and setting

This retrospective comparative observational study was conducted at Shalamar Hospital, Lahore, Pakistan, from March 2023 to March 2026. A total of 305 patients diagnosed with HF were included for analysis. Medical records were reviewed to extract relevant demographic, clinical, laboratory, echocardiographic, and outcome data. Since this was a retrospective record review, all eligible patients meeting the inclusion criteria during the study period were included. Patients were categorized into two groups based on LVEF: HFrEF and HFpEF.

Inclusion and exclusion criteria

Patients aged 18 years or older with a documented diagnosis of HF were included. HF was defined by the presence of compatible clinical symptoms (e.g., dyspnea, fatigue, or exercise intolerance) and/or signs of congestion together with echocardiographic assessment. Patients were required to have a documented LVEF and complete clinical records.

Patients were classified as having HFrEF if LVEF was < 40%. Patients were classified as having HFpEF if LVEF was ≥ 50% and the medical record documented a clinical diagnosis of HFpEF by the treating cardiologist, supported by symptoms and/or signs of HF together with preserved systolic function on echocardiography. Where available, additional echocardiographic findings suggestive of elevated left ventricular filling pressures or structural heart disease, such as left atrial enlargement or diastolic dysfunction, were considered to support the diagnosis. Patients with HF with mildly reduced ejection fraction (HFmrEF; LVEF 40-49%) were excluded because this represents a distinct clinical phenotype with overlapping characteristics. Patients with incomplete or missing medical records, congenital heart disease, severe valvular heart disease, or acute myocardial infarction at presentation were also excluded.

Data collection

Data were collected retrospectively using a structured proforma from hospital medical records, discharge summaries, echocardiography reports, laboratory databases, and diagnostic reports. Demographic variables included age, sex, and BMI. Clinical variables included New York Heart Association (NYHA) functional class at admission, vital signs, and presenting symptoms. Data extraction was independently performed by two investigators using a standardized data collection proforma. Discrepancies were resolved by consensus through review of the original medical records. Inter-rater reliability for key study variables was assessed using Cohen's kappa statistic for categorical variables, with a kappa value ≥ 0.80 indicating excellent agreement.

Comorbidities including hypertension, diabetes mellitus, ischemic heart disease, chronic kidney disease, and atrial fibrillation were identified from documented physician diagnoses, discharge summaries, medication history, and relevant clinical documentation in the medical record. Obesity was defined as a documented BMI ≥ 30 kg/m² where BMI data were available. No independent verification of comorbidities using predefined laboratory or clinical thresholds was performed because of the retrospective study design. NYHA functional class was extracted directly from the clinician-documented assessment at the time of admission. The study investigators did not assign NYHA class retrospectively based on symptom descriptions.

Patients were grouped according to echocardiographic LVEF measurements, with HFrEF defined as LVEF < 40% and HFpEF as LVEF ≥ 50%. Laboratory variables included hemoglobin concentration, renal function tests, and natriuretic peptide levels where available. Echocardiographic variables included LVEF, left atrial size, and diastolic function parameters when documented. Cardiac rhythm abnormalities were identified from admission electrocardiograms, inpatient cardiac monitoring records when available, and documented cardiology diagnoses in the medical record. Patients with a documented history of atrial fibrillation or ventricular arrhythmias were classified accordingly. Because prolonged rhythm monitoring such as Holter monitoring was not routinely available for all patients, paroxysmal arrhythmias may have been underdetected.

The primary outcomes were in-hospital mortality, length of hospital stay, and 30-day readmission. A composite adverse outcome was defined a priori as the occurrence of at least one of the following: in-hospital mortality, prolonged hospital stay (defined as > 7 days), or 30-day readmission. Secondary outcomes included in-hospital complications and functional status at admission based on the documented NYHA classification.

Data analysis

Data were analyzed using SPSS Statistics version 26.0. Continuous variables were expressed as mean ± standard deviation (SD), whereas categorical variables were presented as frequencies and percentages. Continuous variables were compared using the independent-samples t-test, while categorical variables were compared using the chi-square test or Fisher's exact test, as appropriate.

Multivariable binary logistic regression analysis was performed to identify independent predictors of the composite adverse outcome. Variables with a p-value < 0.20 in univariate analyses, together with clinically relevant variables identified a priori, were considered for inclusion in the multivariable model. Adjusted odds ratios (AORs) with 95% confidence intervals (CIs) were reported. A two-sided p-value ≤ 0.05 was considered statistically significant. Because the study was exploratory and hypothesis-generating, no formal adjustment for multiple comparisons was applied. Therefore, findings with p-values close to the significance threshold should be interpreted with caution.

## Results

A total of 305 patients with HF were included in the study. The mean age of the study population was 58.7 ± 13.2 years. Patients with HFpEF were significantly older (62.4 ± 11.8 years) compared to those with HFrEF (56.1 ± 13.7 years). Overall, 178 patients (58.4%) were male and 127 (41.6%) were female. A male predominance was observed in the HFrEF group (116, 63.7%), whereas a relatively higher proportion of females was noted in the HFpEF group (61, 49.6%).

Hypertension was the most prevalent comorbidity, present in 198 patients (64.9%), followed by ischemic heart disease in 186 (61.0%), diabetes mellitus in 161 (52.8%), obesity in 81 (26.6%), and chronic kidney disease in 68 (22.3%) patients. HFpEF patients had a significantly higher prevalence of hypertension (92, 74.8% vs. 106, 58.2%) and diabetes mellitus (75, 61.0% vs. 86, 47.3%) compared to HFrEF patients. In contrast, ischemic heart disease (125, 68.7% vs. 61, 49.6%) and chronic kidney disease (48, 26.4% vs. 20, 16.3%) were more common in the HFrEF group (Table 1).

**Table 1:** Baseline demographic and clinical characteristics Test applied: independent-samples t-test for continuous variables (age) and Chi-square test for categorical variables. Statistical significance: p ≤ 0.05 HFrEF: heart failure with reduced ejection fraction; HFpEF: heart failure with preserved ejection fraction; SD: standard deviation

Variable	Category	HFrEF (n = 182)	HFpEF (n = 123)	Total (n = 305)
Age, years, mean ± SD	—	56.1 ± 13.7	62.4 ± 11.8	58.7 ± 13.2
Gender, n (%)	Male	116 (63.7%)	62 (50.4%)	178 (58.4%)
	Female	66 (36.3%)	61 (49.6%)	127 (41.6%)
Hypertension, n (%)	Yes	106 (58.2%)	92 (74.8%)	198 (64.9%)
	No	76 (41.8%)	31 (25.2%)	107 (35.1%)
Diabetes mellitus, n (%)	Yes	86 (47.3%)	75 (61.0%)	161 (52.8%)
	No	96 (52.7%)	48 (39.0%)	144 (47.2%)
Obesity, n (%)	Yes	39 (21.4%)	42 (34.1%)	81 (26.6%)
	No	143 (78.6%)	81 (65.9%)	224 (73.4%)
Ischemic heart disease, n (%)	Yes	125 (68.7%)	61 (49.6%)	186 (61.0%)
	No	57 (31.3%)	62 (50.4%)	119 (39.0%)
Chronic kidney disease, n (%)	Yes	48 (26.4%)	20 (16.3%)	68 (22.3%)
	No	134 (73.6%)	103 (83.7%)	237 (77.7%)

A significantly higher proportion of HFrEF patients presented with advanced symptoms (NYHA class III-IV) (130, 71.4%) compared to HFpEF patients (65, 52.8%), while milder symptoms (NYHA class I-II) were more frequent in HFpEF (58, 47.2% vs. 52, 28.6%; p = 0.003). Atrial fibrillation was significantly more common in HFpEF patients (36, 29.3% vs. 33, 18.1%; p = 0.028), whereas ventricular arrhythmias were more frequently observed in HFrEF patients (41, 22.5% vs. 14, 11.4%; p = 0.017) (Table 2).

**Table 2:** Clinical presentation and cardiac rhythm abnormalities Test applied: Chi-square test for categorical variables. Statistical significance: p ≤ 0.05 HFrEF: heart failure with reduced ejection fraction; HFpEF: heart failure with preserved ejection fraction; NYHA: New York Heart Association

Variable	Category	HFrEF (n = 182), n (%)	HFpEF (n = 123), n (%)	Total (n = 305), n (%)	P-value
NYHA class	I–II	52 (28.6%)	58 (47.2%)	110 (36.1%)	0.003
	III–IV	130 (71.4%)	65 (52.8%)	195 (63.9%)	—
Atrial fibrillation	Yes	33 (18.1%)	36 (29.3%)	69 (22.6%)	0.028
	No	149 (81.9%)	87 (70.7%)	236 (77.4%)	—
Ventricular arrhythmias	Yes	41 (22.5%)	14 (11.4%)	55 (18.0%)	0.017
	No	141 (77.5%)	109 (88.6%)	250 (82.0%)	—

In-hospital mortality was higher among patients with HFrEF compared to HFpEF (26, 14.3% vs. 10, 8.1%), although this difference did not reach statistical significance (p = 0.089). The mean length of hospital stay was significantly longer in the HFrEF group (7.6 ± 3.2 days) compared to the HFpEF group (5.9 ± 2.7 days; p = 0.001). Similarly, 30-day readmission rates were higher in HFrEF patients (50; 27.5% vs. 23, 18.7%; p = 0.045) (Table 3).

**Table 3:** In-hospital outcomes and readmission Test applied: Chi-square test for categorical variables; independent-samples t-test for the continuous variable (length of stay). Statistical significance: p ≤ 0.05 HFrEF: heart failure with reduced ejection fraction; HFpEF: heart failure with preserved ejection fraction; SD: standard deviation

Variable	Category	HFrEF (n = 182)	HFpEF (n = 123)	Total (n = 305)	P-value
In-hospital mortality, n (%)	Yes	26 (14.3%)	10 (8.1%)	36 (11.8%)	0.089
	No	156 (85.7%)	113 (91.9%)	269 (88.2%)	—
Length of stay, days, mean ± SD	—	7.6 ± 3.2	5.9 ± 2.7	6.9 ± 3.1	0.001
30-day readmission, n (%)	Yes	50 (27.5%)	23 (18.7%)	73 (23.9%)	0.045
	No	132 (72.5%)	100 (81.3%)	232 (76.1%)	—

On multivariate logistic regression analysis, age greater than 60 years (AOR: 1.8; 95% CI: 1.1-2.9; p = 0.021), chronic kidney disease (AOR: 2.3; 95% CI: 1.4-3.8; p = 0.003), and NYHA class III-IV at presentation (AOR: 2.7; 95% CI: 1.6-4.5; p < 0.001) were independently associated with adverse clinical outcomes. HF phenotype was also an independent predictor, with HFrEF patients demonstrating higher odds of adverse outcomes compared to HFpEF patients (AOR: 1.9; 95% CI: 1.2-3.1; p = 0.008). The multivariable logistic regression model demonstrated an adequate fit to the data. The Omnibus Test of Model Coefficients was statistically significant (χ² = 27.84, df = 4, p < 0.001), indicating that the model containing the predictor variables performed significantly better than the null model. The Hosmer-Lemeshow goodness-of-fit test showed no evidence of poor model fit (χ² = 6.21, df = 8, p = 0.624). The model explained approximately 15.8% of the variance in the composite adverse outcome (Nagelkerke R² = 0.158) and correctly classified 72.8% of cases (Table 4).

**Table 4:** Multivariate logistic regression analysis for predictors of adverse outcomes Test applied: multivariate binary logistic regression analysis. Outcome variable: composite adverse outcome (mortality, readmission, or prolonged hospital stay as defined in the study). Statistical significance: p ≤ 0.05. Model performance: Omnibus test χ² = 27.84, df = 4, p < 0.001; Hosmer-Lemeshow χ² = 6.21, df = 8, p = 0.624; Nagelkerke R² = 0.158; overall classification accuracy = 72.8% AOR: adjusted odds ratio; CI: confidence interval; NYHA: New York Heart Association; HFrEF: heart failure with reduced ejection fraction; HFpEF: heart failure with preserved ejection fraction

Variable	AOR	95% CI	P-value
Age > 60 years	1.8	1.1–2.9	0.021
Chronic kidney disease	2.3	1.4–3.8	0.003
NYHA class III–IV	2.7	1.6–4.5	< 0.001
HFrEF (vs. HFpEF)	1.9	1.2–3.1	0.008

## Discussion

This retrospective comparative study evaluated differences in clinical characteristics, short-term outcomes, and predictors of adverse outcomes between patients with HFpEF and HFrEF. The findings demonstrate clear differences in demographic profiles, comorbidity patterns, clinical presentation, and outcomes between the two phenotypes, along with identification of key predictors of poor prognosis. Patients with HFpEF were significantly older and more frequently female compared to those with HFrEF. These findings are consistent with existing literature, which consistently reports HFpEF as a condition predominantly affecting elderly and female populations [9,11]. This pattern is likely related to age-associated vascular stiffening, differences in myocardial remodeling, and a higher burden of comorbidities in these patients. In contrast, HFrEF patients were relatively younger and predominantly male, which may be attributed to the higher prevalence of ischemic heart disease in this group, a well-established major etiological factor for systolic dysfunction [12,13].

Comorbid conditions such as hypertension, diabetes mellitus, and obesity were more prevalent in HFpEF patients. This aligns with previous studies highlighting the central role of metabolic syndrome and systemic inflammation in the pathophysiology of HFpEF [14,15]. These comorbidities contribute to endothelial dysfunction, myocardial stiffness, and impaired ventricular relaxation, which are key mechanisms underlying HFpEF. Conversely, ischemic heart disease was significantly more common among HFrEF patients, reinforcing its established association with myocardial injury, ventricular remodeling, and systolic dysfunction [13,15].

Regarding clinical presentation, a higher proportion of HFrEF patients presented with advanced symptoms (NYHA class III-IV), indicating more severe functional impairment at admission. This finding is consistent with prior studies demonstrating that HFrEF is typically associated with reduced cardiac output and overt volume overload symptoms [16,17]. In contrast, HFpEF patients often present with less severe symptoms despite significant functional limitation, which may delay diagnosis and contribute to under-recognition of disease severity [16,18]. In terms of rhythm abnormalities, atrial fibrillation was more frequent in HFpEF patients, whereas ventricular arrhythmias were more common in HFrEF patients. These findings are physiologically plausible, as atrial structural remodeling and elevated left atrial pressures in HFpEF predispose to atrial fibrillation, while ventricular dilation and myocardial scarring in HFrEF increase susceptibility to ventricular arrhythmias [11,14].

With respect to clinical outcomes, HFrEF patients demonstrated worse short-term outcomes compared to HFpEF patients. Although in-hospital mortality was numerically higher in the HFrEF group, the difference did not reach statistical significance. Because this retrospective study included all eligible patients during the study period rather than a prospectively determined sample size, the study may have been underpowered to detect modest differences in mortality. Therefore, the absence of statistical significance should not necessarily be interpreted as evidence of no true difference between the two groups. However, HFrEF patients had significantly longer hospital stays and higher 30-day readmission rates, indicating a greater overall healthcare burden. These findings are consistent with previous studies reporting more complicated clinical courses and increased resource utilization in HFrEF populations [13,18,19].

The multivariate analysis identified age greater than 60 years, chronic kidney disease, NYHA class III-IV, and HFrEF phenotype as independent predictors of adverse outcomes. These findings are supported by existing literature, which consistently identifies advanced age, renal dysfunction, and higher functional class as strong determinants of poor prognosis in HF patients [19,20]. The association of HFrEF with adverse outcomes further emphasizes the clinical severity of systolic dysfunction compared to HFpEF in the short-term setting. The clinical implications of this study are significant. Differentiating between HFpEF and HFrEF enables better risk stratification and more individualized management strategies. Patients with HFpEF require aggressive management of comorbidities such as hypertension, diabetes, and obesity, which play a central role in disease progression [14,16]. In contrast, patients with HFrEF require early initiation and optimization of guideline-directed medical therapy to improve outcomes and reduce hospitalizations [20,21].

Limitations

This study has several limitations. First, the retrospective design limits causal inference and is subject to missing or incomplete data inherent to medical record-based studies. Second, being a single-center study, the findings may have limited generalizability to broader populations with different demographic and clinical characteristics. Third, the exclusion of patients with HFmrEF may have resulted in the loss of clinically relevant transitional data between HFpEF and HFrEF. Fourth, certain important variables, including natriuretic peptide levels and comprehensive echocardiographic parameters required for the contemporary guideline-based diagnosis of HFpEF (such as E/e′ ratio, left atrial volume index, and tricuspid regurgitation velocity), were not consistently available due to the retrospective nature of the study. Consequently, HFpEF classification was based on documented clinical diagnosis, preserved LVEF (≥50%), and available echocardiographic findings, which may have resulted in some degree of misclassification and limited more detailed phenotypic characterization.

Additionally, the primary multivariable analysis used a composite adverse outcome comprising in-hospital mortality, prolonged hospital stay, and 30-day readmission. Because prolonged hospital stay and readmission were also analyzed as individual outcomes and differed significantly between HF phenotypes, some degree of circularity cannot be excluded when interpreting the association between HF phenotype and the composite endpoint. In addition, no formal correction for multiple comparisons was performed. Consequently, statistically significant findings with p-values close to 0.05 may be more susceptible to type I error and should be interpreted cautiously. Furthermore, some clinical outcomes were based on routine clinical documentation rather than standardized outcome assessment tools, introducing potential measurement bias. Finally, the study focused only on short-term outcomes, including in-hospital mortality and 30-day readmission, and did not evaluate long-term survival, functional status, or quality of life.

## Conclusions

In this retrospective comparative study, patients with HFrEF presented with more advanced symptoms and experienced significantly longer hospital stays and higher 30-day readmission rates than patients with HFpEF. Although in-hospital mortality was numerically higher among patients with HFrEF, the difference was not statistically significant. Patients with HFpEF were older and had a higher prevalence of hypertension, diabetes mellitus, and obesity, reflecting the differing demographic and comorbidity profiles of the two heart failure phenotypes. Overall, these findings demonstrate distinct clinical characteristics and short-term outcome patterns between HFrEF and HFpEF. Given the retrospective single-center design and the study's methodological limitations, these findings should be interpreted with appropriate caution. Larger prospective, multicenter studies using standardized guideline-based diagnostic criteria are warranted to validate these observations and further refine phenotype-specific risk stratification and management strategies in patients with HF.
